# Safety and efficacy of rituximab in refractory pediatric Systemic Lupus Erythematosus nephritis: a single-center experience of northern Greece

**DOI:** 10.1186/1546-0096-9-S1-P246

**Published:** 2011-09-14

**Authors:** M Trachana, A Koutsonikoli, E Farmaki, N Printza, V Tzimouli, F Papachristou

**Affiliations:** 1 First Department of Pediatrics, Hippokration General Hospital, Aristotle University, Thessaloniki, Greece

## Background

Lupus nephritis (LN) is the major determinant of outcome in pediatric Systemic Lupus Erythematosus (pSLE) and its treatment remains a challenge. Aim. To report the experience of our centre in treating with Rituximab (RTX) SLE patients with severe LN.

## Methods

Four pSLE patients with, refractory to conventional immunosuppressive treatment, biopsy-proven LN received four doses of 375-500mg/m^2^ RTX, 2-3 weeks apart. All patients were concurrently on corticosterois (CSs) and mycophenolate mofetil. Patients’ clinical and laboratory findings were recorded at RTX initiation, after each infusion and at 3.4±2.1 month-intervals thereafter. pSLE activity was assessed using the European Consensus Lupus Activity Measurement (ECLAM), while LN activity using 24-hour urine protein excretion and serum cystatin C.

## Results

Patients were followed-up for 6-21 months (median: 16 months). Full Β cell depletion was noticed 2-4 weeks after RTX initiation and lasted 4-7 months. All patients achieved complete LN remission 3.5 months (range: 2-4) after RTX initiation which was retained in 3 patients through the follow-up period. One patient relapsed 15 months after RTX initiation and received one additional RTX dose. ECLAM scores and CSs doses were markedly reduced in all patients, while complement levels increased. No side effects or infections were observed.

## Conclusion

RTX is an alternative, safe and efficient treatment for refractory LN.

